# High expression of IQGAP3 promotes the infiltration of M0 macrophages into the TME, resulting in a poor prognosis for gastric cancer patients

**DOI:** 10.1093/gastro/goaf095

**Published:** 2025-12-23

**Authors:** Shurong Liu, Hongwei Zhang, Bignqi Dong, Xiaocong Pang, Yong Jiang, Guowei Chen, Yingchao Wu, Tao Liu, Changyou Wang, Junling Zhang, Xin Wang

**Affiliations:** Department of Gastrointestinal Surgery, Peking University First Hospital, Beijing, P. R. China; Department of Gastrointestinal Surgery, Peking University First Hospital, Beijing, P. R. China; Department of Colorectal Cancer, Tianjin Medical University Cancer Institute & Hospital, National Clinical Research Center for Cancer, Tianjin’s Clinical Research Center for Cancer, Tianjin Key Laboratory of Cancer Prevention and Therapy, Tianjin, P. R. China; Department of Gastrointestinal Surgery, Peking University First Hospital, Beijing, P. R. China; Department of Pharmacy, Peking University First Hospital, Beijing, P. R. China; Department of Gastrointestinal Surgery, Peking University First Hospital, Beijing, P. R. China; Department of Gastrointestinal Surgery, Peking University First Hospital, Beijing, P. R. China; Department of Gastrointestinal Surgery, Peking University First Hospital, Beijing, P. R. China; Department of Gastrointestinal Surgery, Peking University First Hospital, Beijing, P. R. China; Department of Gastrointestinal Tumor Surgery, North China University of Science and Technology Affiliated Hospital, Tangshan, Hebei, P. R. China; Department of Gastrointestinal Surgery, Peking University First Hospital, Beijing, P. R. China; Department of General Surgery, Peking University Health Science Center & Colorectal Cancer Laboratory of PKU FH, Beijing, P. R. China; Department of Gastrointestinal Surgery, Peking University First Hospital, Beijing, P. R. China

**Keywords:** gastric cancer, tumor microenvironment, IQGAP3, M0 macrophages

## Abstract

**Background:**

The tumor microenvironment (TME) plays an important role in regulating gastric cancer (GC) progression. The infiltration of M0 macrophages into the TME negatively affects the prognosis of patients with various tumors.

**Methods:**

The data were obtained from the TCGA and GEO databases and our hospital (107 patients). The expression of IQGAP3 was knocked down in AGS and NCI-N87 GC cells. Cell proliferation, migration, and invasion assays were performed. RNA sequencing was performed on GC cells with different IQGAP3 expression. AGS and THP-1 cells were mixed to create a co-cultured subcutaneous tumor model in nude mice. Tumor growth in the mice was observed by using luciferin fluorescence and the tumor tissues were subjected to immunohistochemistry.

**Results:**

The expression of IQGAP3 was increased in GC tissues (*P *< 0.001) and was associated with infiltration of M0 macrophages and a poor prognosis for GC patients (*P *< 0.01). Knockdown of IQGAP3 resulted in decreased expression of CXCL13 (*P *< 0.001) and less phosphorylation of pSTAT3 and pERK1/2 (*P *< 0.001). The expression of CXCL13 was decreased after the pSTAT3 and pERK1/2 phosphorylation inhibitors were added. In the co-culture experiment, the M0/THP-1 ratio decreased significantly in the low-IQGAP3-expression group (*P *< 0.001). However, adding recombinant human CXCL13 proteins to the low IQGAP3 expression group increased the M0/THP-1 ratio. *In vivo*, tumor growth and M0 macrophage infiltration were both suppressed in the group with low IQGAP3 expression.

**Conclusion:**

IQGAP3 is a potential pro-carcinogenic factor in GC. IQGAP3 promotes the expression and secretion of CXCL13 via the ERK1/2 and STAT3 pathways, thereby causing M0 macrophages to infiltrate the TME.

## Introduction

Gastric cancer (GC) has the fifth-highest incidence rate and the third-highest mortality rate globally. Its progression is highly dependent on the dynamic regulation of the tumor microenvironment (TME) [1, 2]. The infiltration of immune cells into the TME—particularly tumor-associated macrophages (TAMs)—drives GC malignant progression by promoting angiogenesis, immunosuppression, and metastasis [3]. Recent studies have found that unpolarized M0 macrophages are highly prevalent in tumors such as glioma and hepatocellular cancer, resulting in a poor prognosis for patients [4, 5].

IQGAP3 is a key regulator of the Ras/ERK pathway [6, 7] and is highly expressed in various cancers, such as hepatocellular and lung cancers, in which it promotes tumor proliferation and invasion [8, 9]. In GC, IQGAP3 accelerates disease progression by enhancing the capacity of cells to migrate [8], but it is not known whether it influences the progression of GC by modulating the immune cells of the TME.

Inflammation is a key factor in promoting tumor progression in the TME [10]. TAMs including M0, M1, and M2 macrophages play a crucial role in regulating tumor growth, angiogenesis, migration, invasion, and metastasis [3, 11–13].

## Methods

### Data sources

Data were collected from the TCGA and GEO (GSE15459, GSE62254, and GSE51105) databases. Patients were divided into two groups based on their median IQGAP3 expression. In the gene co-expression analysis, normalized expression data underwent quality-control filtering. Pairwise Spearman’s rank correlations (*ρ*) were computed across all samples to capture nonlinear relationships. Pan-cancer analysis of IQGAP3 was performed by using the TIMER2.0 website (cistrome.org). The CCLE database was used to check the IQGAP3 expression in different GC cell lines. The K-M Plotter database was used for survival analysis.

### Clinical data

A total of 107 GC patients were recruited from Peking University First Hospital between 2016 and 2018. The patients comprised 73 males and 34 females, with an average age of 58.36 years. The inclusion criteria were patients with (i) a pathological diagnosis of gastric adenocarcinoma and (ii) no prior chemotherapy, immunotherapy, or surgery. The exclusion criteria were patients with (i) other malignant tumors; (ii) incomplete tumor node metastasis classification (TNM) staging information; and (iii) incomplete patient survival information. Informed consent was obtained from all patients. This study was approved by the Ethics Committee of Peking University First Hospital.

### Immunohistochemical staining

The GC tissues were preserved in paraffin. After slicing, the sections were deparaffinized and rehydrated. Then, the sections were incubated overnight at 4°C with IQGAP3, CXCL13, CD86, CD163 (1:1,000, Abcam, Cambridge, UK), CD14, and CD68 (1:1,000, Cell Signaling Technology, Massachusetts, USA) antibodies. The tissues were then incubated with a secondary antibody, after which diaminobenzidine (DAB) was used for visualization. The immunohistochemical (IHC) results were evaluated by using a staining-intensity score and a percentage-of-positive-cells score. There are four levels of the staining-intensity score, as follows: negative (0), light yellow (1), yellow (2), and brown (3). There are three levels of the positive-cell-proportion score: staining in 6%–25% of cells (1); staining in 26%–50% of cells (2); and staining in >50% of cells (3). Total scores of <5 were categorized as low-expression, while others were categorized as high-expression.

### ELISA

The expression of CXCL13 by tumor cells was measured by using an enzyme-linked immunosorbent assay (ELISA) kit (ELK Biotechnology, Wuhan, China). The color change was measured spectrophotometrically at a wavelength of 450 nm. The concentration of CXCL13 in the samples was determined by comparing their optical density with that of the standard curve.

### Cell-line culture

GC cell lines (AGS, SGC-7901, HGC-27, NCI-N87, and BGC-823) were obtained from the Cancer Institute of the Chinese Academy of Medical Sciences. THP-1 and GES-1 were purchased from the Global Bioresource Centre (ATCC). Cells were cultured under optimal conditions in RPMI-1640 medium supplemented with 10% fetal bovine serum (Sigma Life Science, Merck KGaA, Darmstadt, Germany).

### Cell-transfection assay

The knocking-down of IQGAP3 was achieved by using shRNA plasmids that were marked by using green fluorescent protein (GFP) and puro (Suzhou Gemma Genetics, Jiangsu, China) with the following sequences: IQGAP3-sh1: GCGGCAGAATGTTGCCTATCA; IQGAP3-sh2: CAGCTGTGGTCCTGATTAACC; and IQGAP3-sh3: CCAGCAGACACAGCTTTCTGGG. The plasmid with the highest transfection efficiency was selected for the experiment. For shRNA plasmid transfection, adherent cells were seeded in a culture plate to reach 70%–90% confluency. Transfection complexes were prepared by mixing the plasmid DNA with a lipofectamine-based reagent in a serum-free medium, incubating for 15 min at room temperature, then replacing the cell culture medium and adding the complexes dropwise. After 24 h of incubation (37°C, 5% CO_2_), the transfection efficiency was assessed via GFP fluorescence microscopy. Selection was initiated by replacing the medium with puromycin-containing medium to eliminate non-transfected cells [14].

### Cell-proliferation assay

Cell proliferation was examined by using the Cell Counting Kit-8 (Bimake, Shanghai, China). AGS cells were divided into two groups according to IQGAP3 expression levels; 5,000 cells with normal or reduced IQGAP3 expression were seeded into 96-well plates, each with 5 replicate wells.

### Invasion assay

The invasion assay was performed by using Transwell chambers pre-coated with Matrigel (membrane pore size of 8 µm; Corning, MA, USA). Then, 500 µL of filtered conditioned media from the GC cell lines was added to the lower chamber and 1 × 10^4^ GC cells in 200 µL of serum-free RPMI-1640 were plated into the upper chamber. The chamber was incubated for 36 h at 37°C and the number of invaded cells was quantified by counting four random fields per filter. All assays were performed in triplicate [15].

### Cell-colony-formation assay

AGS cells (1 × 10³) were cultured in six-well plates for 14 days and the formation of colonies was subsequently monitored by using a phase-contrast light microscope. Five fields per sample were photographed by using a digital camera. ImageJ software (Version 1.49, National Institutes of Health, MD, USA) was used to assess the number of colonies (>50 μm). All assays were performed in triplicate.

### Western-blotting assay

Total cell lysates were prepared and analysed by using Western blotting. After the protein concentration of each sample was adjusted, sodium dodecyl sulfate (SDS)-polyacrylamide gel electrophoresis was performed to separate the different proteins. The following antibodies were used: IQGAP3 (1:1,000, Abcam); p-ERK1/2 and ERK1/2; p-STAT3 and STAT3; and CXCL13 and β-actin (all 1:1,000, Cell Signaling Technology). These were then incubated overnight at 4°C. The electrochemiluminescence (ECL) assay system and Syngene’s GeneGenius gel imaging system were then employed to detect the levels of the target proteins.

### Rescue experiments and concentration-dependent experiments

The STAT3 and ERK1/2 phosphorylation inhibitors BBI608 and GDC-0994 were added to the negative control (NC) group and then the expression level of the CXCL13 protein was measured by using Western blotting. Concentration-dependent experiments were performed based on the NC group.

### Indirect cell co-culture and flow-cytometry assay

The conditioned medium from AGS cells was collected and filtered, and then used to culture THP-1 cells. To confirm whether the differentiation of THP-1 cells was affected by CXCL13, varying concentrations of rh-CXCL13 protein were added to the IQGAP3-sh1 group. THP-1 differentiation levels were detected by using a flow cytometer. The transformation rate was represented by the ratio of M0 macrophages to THP-1 cells.

### Tumor xenograft models in mice

AGS cells (2 × 10^6^) containing the luciferase gene were mixed with THP-1 cells in equal proportions and injected into BALB/c nude mice subcutaneously (Charles River, Beijing, China). The tumor volume was calculated by using the following formula: Volume = 0.52 × width^2^ × length [14]. The mice were injected with firefly D-luciferin and photographed every 3 days by using the Spectrum Living Image 4.0 system before the tumor diameter reached 1.5 cm [15].

### Statistical analysis

Statistical analyses were performed by using R Studio version 4.2.0, with a *P* value of <0.05 indicating a significant correlation. Images were created by using GraphPad Prism and R software. A Student’s *t*-test was used to calculate the correlation between the two sets of data. Other data were categorical variables, for which we counted the number directly. Depending on the size of the data sample, we used chi-squared or Fisher’s exact tests to calculate the correlation between the two sets of data.

The results were shown as *P* values: *P < 0.05, **P < 0.01, ***P < 0.001. The *t*-test and paired difference analysis were employed for the differential analysis of IQGAP3 in tumor and normal tissues. Survival analysis was performed by using the Kaplan–Meier Plotter database (https://kmplot.com/analysis/). Logistic regression was used for the correlation analysis. The infiltration of 22 immune cells into the gastric TME was assessed by using CIBERSORT [20]. The GEPIA and GeneCards websites (https://gepia.cancer and https://www.genecards.org/) were used to validate the gene expressions in GC.

## Results

### The expression of IQGAP3 was significantly higher in GC and was associated with a worse prognosis

**Figure 1.**
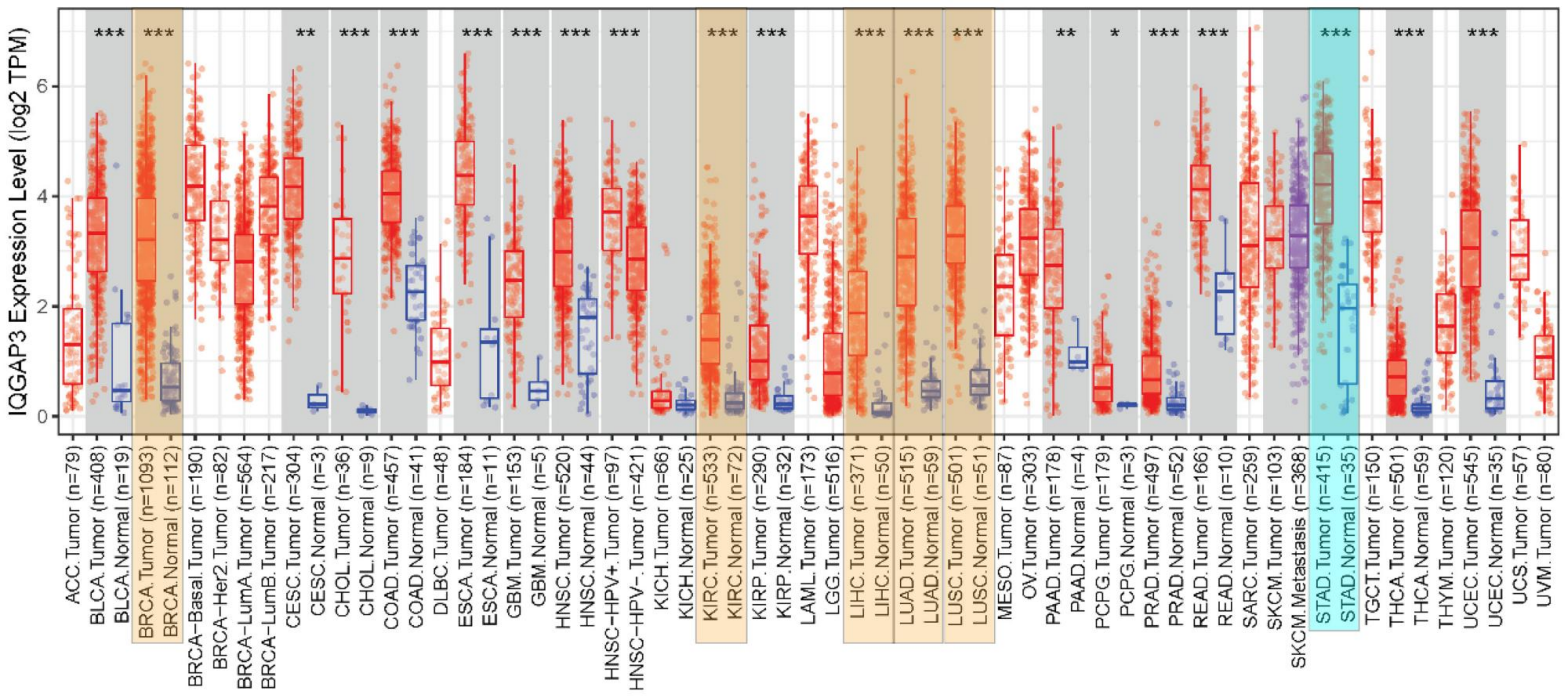
Pan-cancer analysis of IQGAP3 in different normal tissues and tumors. The top five tumor types with significant differences in IQGAP3 expression are breast invasive carcinoma (BRCA, *P* = 3.01E^−60^), lung adenocarcinoma (LUAD, *P* = 7.66E^−34^), kidney renal clear cell carcinoma (KIRC, *P* = 1.66E^−31^), lung squamous cell carcinoma (LUSC, *P* = 4.86E^−31^), and liver hepatocellular carcinoma (LIHC, *P* = 9.12E^−26^). Stomach adenocarcinoma (STAD) ranked ninth (*P* = 2.25E^−19^). IQGAP3 expression was upregulated in all of these tumors.

**Figure 2.**
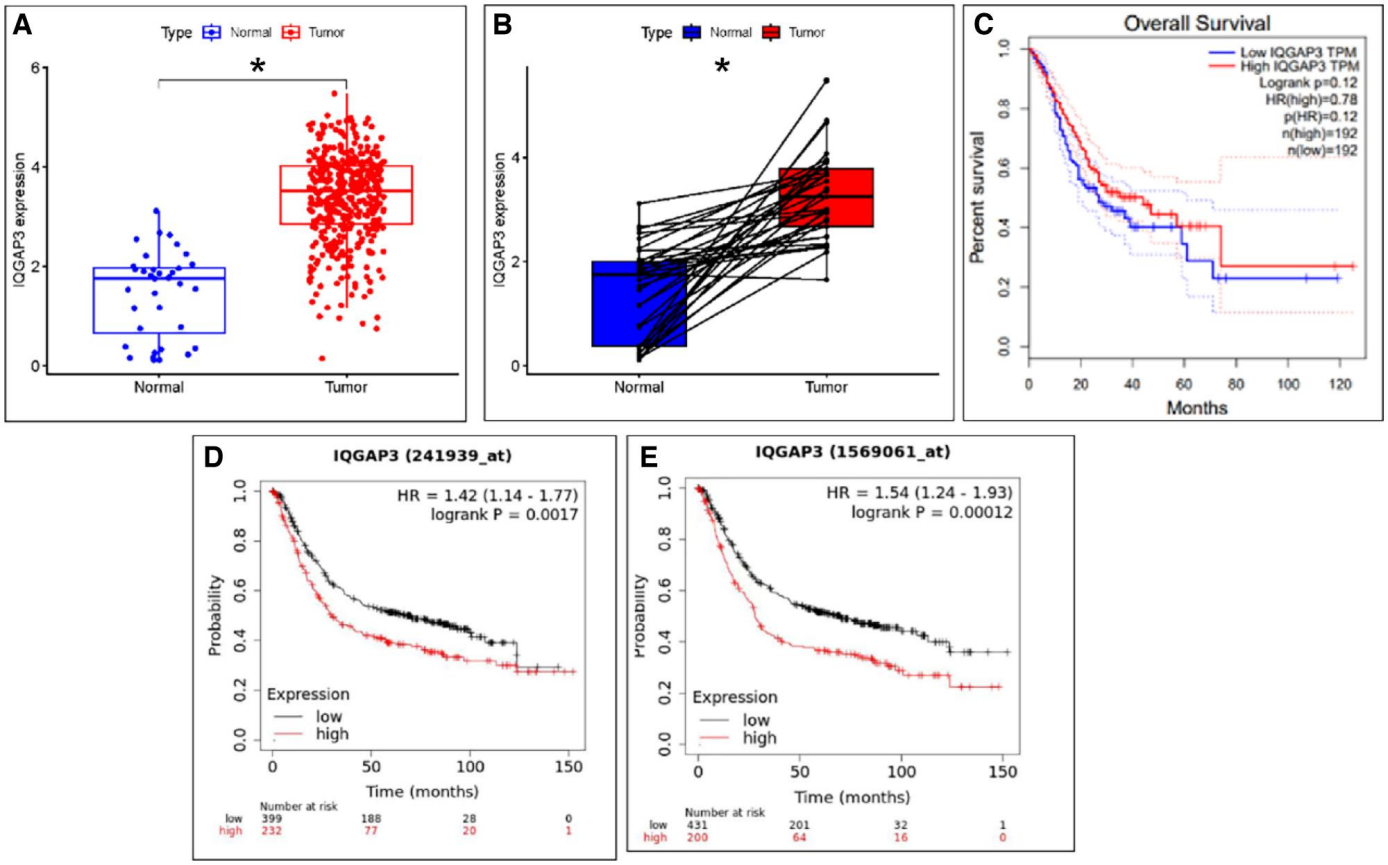
The expression of IQGAP3 was upregulated in GC tissue and associated with a poor prognosis for patients. (A) Our analysis of the differences in IQGAP3 expression between normal and tumor tissues from the TCGA database showed that the level of IQGAP3 expression was significantly higher in GC tissues. (B) Paired difference analysis of IQGAP3 between normal and tumor tissues. The box plot shows IQGAP3 mRNA expression. (C) Survival analysis of STAD patients in the TCGA database. There was no significant difference between the high- and low-expression groups. (D, E) Survival analysis of STAD patients was performed by using two IQGAP3 gene probes (IQGAP3 1569061_at and IQGAP3 241939_at) on the K-M website. Patients with high IQGAP3 expression had worse survival outcomes. The bar represents the median expression of tumors or normal tissues, and the lower and upper box ends represent the 25th- and 75th-percentile expression. *P < 0.05, **P < 0.01, ***P < 0.001, based on Student’s *t*-test.

The mRNA expression levels of IQGAP3 in 39 different tumor tissues and 21 normal tissues are shown in Figure 1. The top five tumor types that showed significant differences in IQGAP3 expression were breast cancer, lung adenocarcinoma, kidney renal clear cell carcinoma, lung squamous cell carcinoma, and liver hepatocellular carcinoma. Notably, stomach adenocarcinoma (STAD), which was ranked at ninth, also exhibited increased IQGAP3 expression. The TCGA database revealed that IQGAP3 expression was higher in tumor tissue than in normal tissue (Figure 2A, *P *< 0.001). The paired difference analysis of IQGAP3 expression levels in normal and tumor tissues revealed similar upregulation of IQGAP3 expression in tumor tissues (Figure 2B, *P *< 0.001). The prognostic analysis revealed no significant differences in survival outcomes in the TCGA database (Figure 2C, *P *= 0.12). Nevertheless, two IQGAP3 gene probes in the K-M Plotter database indicated that GC patients with high IQGAP3 expression levels had a worse prognosis (Figure 2D and E, *P *< 0.01).

### IQGAP3 was correlated with high number of M0 macrophages infiltrating the TME

**Figure 3.**
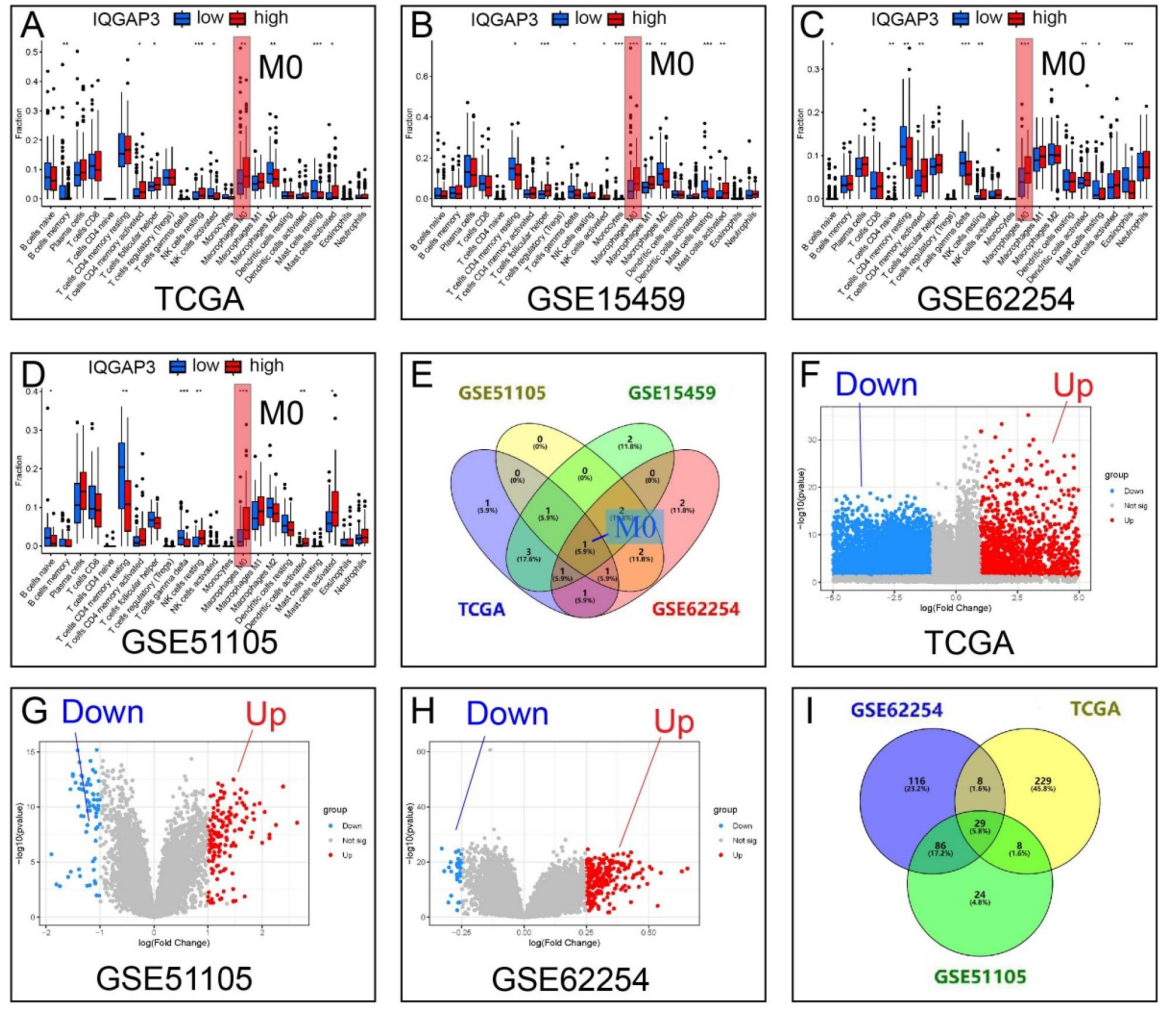
Proportions of 22 subpopulations of immune cells and volcano maps of IQGAP3. (A–D) The TCGA, GSE15459, GSE62254, and GSE51105 databases were used. The red box marks the intersection of the four databases. The results showed that the high-IQGAP3-expression group had more M0 macrophages. (E) Venn diagrams for the four databases. M0 macrophage infiltration was significantly higher in the group with high IQGAP3 expression. (F–H) Gene co-expression analysis was performed in the TCGA, GSE51105, and GSE62254 databases to find genes associated with IQGAP3. According to the median IQGAP3 expression, genes were divided into high- and low-expression groups. Red represents upregulated genes and blue represents downregulated genes. (I) Venn diagram shows the intersection of the three databases and includes 29 genes. A total of 29 upregulated genes can be found in Supplementary Table S1. *P < 0.05, **P < 0.01, ***P < 0.001, based on Student’s *t*-test.

Immune-infiltration analyses were performed by using four separate databases (TCGA, GSE15459, GSE62254, and GSE51105; Figure 3A–D). M0 macrophages were the only significant infiltrators in all the databases (Figure 3E).

### High expression of IQGAP3 was associated with increased expression of CXCL13

Gene co-expression analysis and volcano maps were generated based on the expression of IQGAP3 in the three databases (Figure 3F–H). A total of 29 upregulated genes were found (Supplementary Table 1). Their descriptions are shown in Supplementary Table 2. In GC tissues, three genes were found to be upregulated, 12 were found to be downregulated, and 14 showed no significant change in expression. Of the three upregulated genes, CXCL13 was found to affect the TME.

### Clinical characteristics of 107 GC patients

This study included 107 patients with GC (Table 1), of whom 73 were male (68.2%) and 34 were female (31.8%). The majority of patients were >60 years of age (63.6%). The primary tumor locations were the gastric antrum (36.4%), the body (19.6%), the angle (15.9%), and the cardiac region (28.0%). The diameter of most tumors was ≥5 cm (43.9%) and most tumors exhibited poor differentiation (76.6%).

**Table 1.** Clinicopathological features of 107 cases with GC.

	Number (*n*)	IQGAP3 expression		*P* value	CD14 expression		*P* value	CD68 expression		*P* value	CXCL13 expression		*P* value
		Low	High		Low	High		Low	High		Low	High	
**Sex**													
Male	73 (68.2%)	39	34	0.370	48	25	0.688	58	15	0.102	53	20	0.599
Female	34 (31.8%)	15	19		21	13		22	12		23	11	
**Age (years)**													
>60	68 (63.6%)	34	34	0.898	46	22	0.367	53	15	0.318	54	14	**0.012**
≤60	39 (36.4%)	20	19		23	16		27	12		22	17	*
**Tumor location**													
Gastric antrum	39 (36.4%)	16	23	0.505	29	10	0.074	30	9	0.155	29	10	0.179
Gastric body	21 (19.6%)	12	9		13	8		14	7		12	9	
Gastric angle	17 (15.9%)	10	7		13	4		16	1		15	2	
Gastric cardia	30 (28.0%)	16	14		14	16		20	10		20	10	
**Tumor size (cm)**													
<5	60 (56.1%)	33	27	0.289	41	19	0.347	45	15	0.95	41	19	0.488
≥5	47 (43.9%)	21	26		28	19		35	12		35	12	
**Histological type**													
Adenocarcinoma	67 (62.6%)	29	38	0.151	45	22	0.718	51	16	0.841	46	21	0.766
Signet-ring cell carcinoma	31 (29.0%)	19	12		19	12		22	9		23	8	
Others	9 (8.4%)	6	3		5	4		7	2		7	2	
**Grade**													
Well and moderately differentiated	25 (23.4%)	15	10	0.276	18	7	0.370	20	5	0.491	19	6	0.531
Poorly differentiated	82 (76.6%)	39	43		51	31		60	22		57	25	
**Tumor**													
T1	22 (20.6%)	16	6	0.138	21	1	**<0.001**	22	0	**<0.001**	22	0	**0.004**
T2	11 (10.3%)	5	6		10	1	***	10	1	***	9	2	**
T3	32 (29.9%)	14	18		18	14		24	8		20	12	
T4	42 (39.3%)	19	23		20	22		24	18		25	17	
**Node**													
N0	40 (37.4%)	28	12	**0.004**	34	6	**<0.001**	38	2	**<0.001**	35	5	**0.011**
N1	10 (9.3%)	6	4	**	10	0	***	9	1	***	8	2	*
N2	20 (18.7%)	5	15		9	11		13	7		13	7	
N3	37 (34.6%)	15	22		16	21		20	17		20	17	
**Metastasis**													
M0	104 (97.2%)	52	52	0.569	68	36	0.253	79	25	0.094	74	30	0.866
M1	3 (2.8%)	2	1		1	2		1	2		2	1	
**Clinical stage**													
I	26 (24.3%)	17	9	0.108	25	1	**<0.001**	26	0	**<0.001**	26	0	**0.002**
II	20 (18.7%)	12	8		14	6	***	16	4	***	14	6	**
III	58 (54.2%)	23	35		29	29		37	21		34	24	
IV	3 (2.8%)	2	1		1	2		1	2		2	1	
**Laboratory examination (mean ± SD, number)**													
D-Dimer (mg/mL)	0.26 ± 0.04 (97)	0.23 ± 0.05 (49)	0.29 ± 0.08 (48)	0.539	0.19 ± 0.03 (61)	0.39 ± 0.11 (36)	**0.026** *	0.21 ± 0.03 (73)	0.43 ± 0.14 (24)	**0.034** *	0.26 ± 0.06 (68)	0.28 ± 0.07 (29)	**0.026** *
HGB (g/L)	103.97 ± 2.62 (31)	106.20 ± 3.43 (15)	101.87 ± 3.97 (16)	0.419	103.25 ± 3.17 (16)	104.73 ± 4.34 (15)	0.783	103.85 ± 2.99 (20)	104.18 ± 5.22 (11)	0.953	105.37 ± 3.13 (19)	101.75 ± 4.73 (12)	0.783
CEA (ng/mL)	4.45 ± 0.72 (97)	3.99 ± 0.87 (49)	4.93 ± 1.16 (48)	0.520	3.11 ± 0.41 (62)	6.84 ± 1.81 (35)	**0.012** *	4.06 ± 0.70 (75)	5.81 ± 2.14 (22)	0.312	3.68 ± 0.65 (70)	6.46 ± 1.96 (27)	**0.012** *
CA-199 (U/mL)	26.22 ± 6.59 (92)	21.29 ± 7.83 (45)	30.93 ± 10.53 (47)	0.468	22.72 ± 6.12 (60)	32.79 ± 15.18 (32)	0.470	26.49 ± 7.71 (71)	25.30 ± 12.75 (21)	0.940	21.86 ± 5.43 (68)	38.58 ± 20.1 (24)	0.470
CA-724 (U/mL)	6.02 ± 1.14 (96)	3.76 ± 1.06 (48)	8.29 ± 1.99 (48)	**0.047** *	4.06 ± 0.89 (62)	9.61 ± 0.30 (34)	**0.020** *	4.34 ± 0.86 (74)	11.69 ± 3.90 (22)	**0.006** * *	6.43 ± 1.46 (70)	4.92 ± 1.53 (26)	**0.020** *
AFP (ng/mL)	2.59 ± 0.31 (27)	2.35 ± 0.49 (13)	2.82 ± 0.39 (14)	0.457	2.53 ± 0.37 (16)	2.69 ± 0.54 (11)	0.809	2.52 ± 0.30 (20)	2.81 ± 0.87 (7)	0.685	2.66 ± 0.34 (19)	2.44 ± 0.68 (8)	0.809
**Surgical method**													
Billroth I/II	54 (50.5%)	30	24	0.164	41	13	**0.013** *	46	8	**0.039** *	41	13	0.059
Total gastrectomy	51 (49.5%)	24	29		28	25		34	19		35	18	
Chemotherapy													
Yes	65 (60.7%)	30	35	0.267	38	27	0.105	48	17	0.785	41	24	**0.024** *
No	42 (39.3%)	24	18		31	11		32	10		35	7	

* *P *< 0.05,

** *P *< 0.01,

*** *P *< 0.001.

Advanced disease was prevalent (T4 stage: 39.3%; N3 stage: 34.6%; clinical stage III: 54.2%). Laboratory analysis revealed mean values of 6.02 U/mL for CA-724, 4.45 ng/mL for CEA, and 0.26 mg/mL for D-dimer. The adjuvant chemotherapy rate was 60.7%.

**Figure 4.**
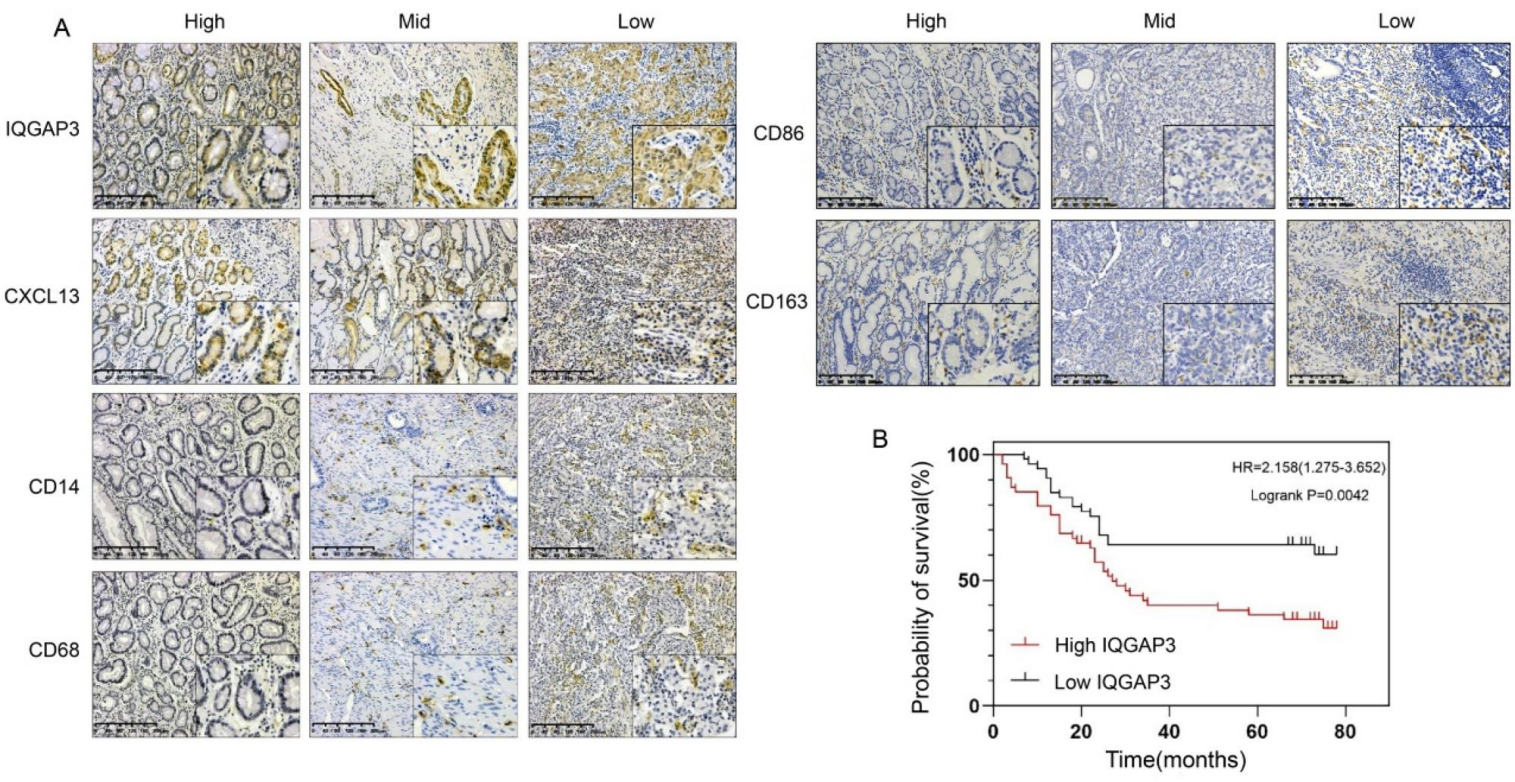
High expression of IQGAP3 was observed in GC tumor tissues and was associated with a poor prognosis. (A) The GC tissues were divided into three groups based on their level of differentiation. These were the high-, intermediate-, and low-differentiation groups. The tissues were stained by using antibodies for LQGAP3, CXCL13, CD14, CD68, CD86, and CD163 (magnification: 10× for the main image and 20× for the inset). There was no significant difference in the expression of CD86 or CD163 between differentially differentiated tissues. (B) Compared with GC patients with low IQGAP3 expression, those with high IQGAP3 expression had a worse prognosis (*P *< 0.01, hazard ratio (HR) = 2.158).

IHC staining was performed on GC tissue samples from these patients (Figure 4A). The survival curve (Figure 4B) showed that patients with high IQGAP3 expression had a worse prognosis (*P *= 0.0042). Table 1 shows a positive correlation between IQGAP3 expression and N stage (*P *= 0.004) and CA-724 level (*P *= 0.047).

The expression of CD14 and CD68 was significantly correlated with the T stage (*P *< 0.001), N stage (*P *< 0.001), clinical stage (*P *< 0.001), D-dimer (P < 0.05), CA-724 (*P *< 0.05), and surgical method (*P *< 0.05). The expression of CXCL13 was significantly correlated with the T stage (*P *= 0.004), N stage (*P *= 0.011), clinical stage (*P *< 0.01), D-dimer *(P *< 0.05), CA-724 (*P *< 0.05), and adjuvant chemotherapy (*P *< 0.05).

### IQGAP3 regulated the JAK–STAT and MAPK pathways

**Figure 5.**
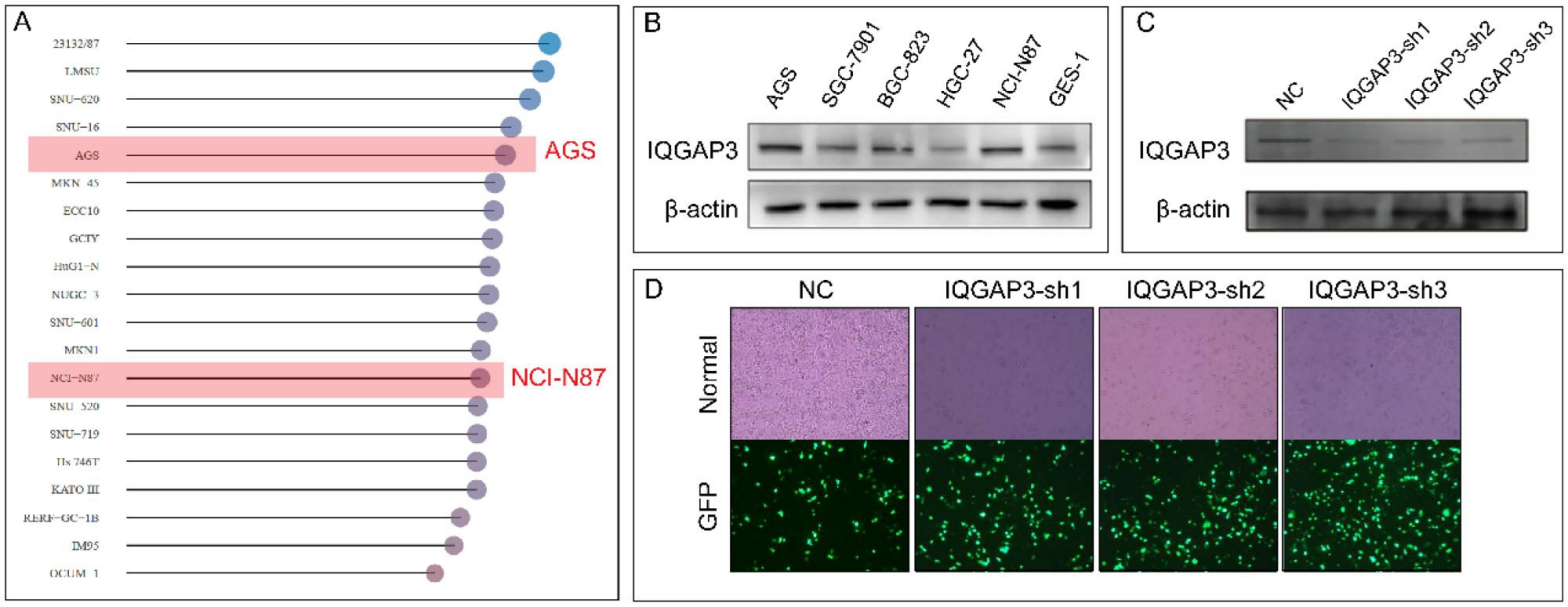
Results of cell selection and cell transfection. (A) Expression levels of IQGAP3 in tumor cell lines in the CCLE database. Among the GC cell lines, AGS and NCI-N87 exhibited high IQGAP3 expression. (B) Expression levels of IQGAP3 in five GC cell lines and GSE-1. IQGAP3 expression was high in the AGS and NCI-N87 cell lines and low in the HGC-27 cell line. (C) Three plasmids (IQGAP3-sh1, IQGAP3-sh2, and IQGAP3-sh3) were used to reduce the expression of IQGAP3 in AGS cells. Western-blotting analysis showed that IQGAP3-sh1 was the most effective. (D) Transfection results for the NC and GFP groups were observed under a 100× microscope. The transfection efficiency of all three plasmids exceeded 70%.

**Figure 6.**
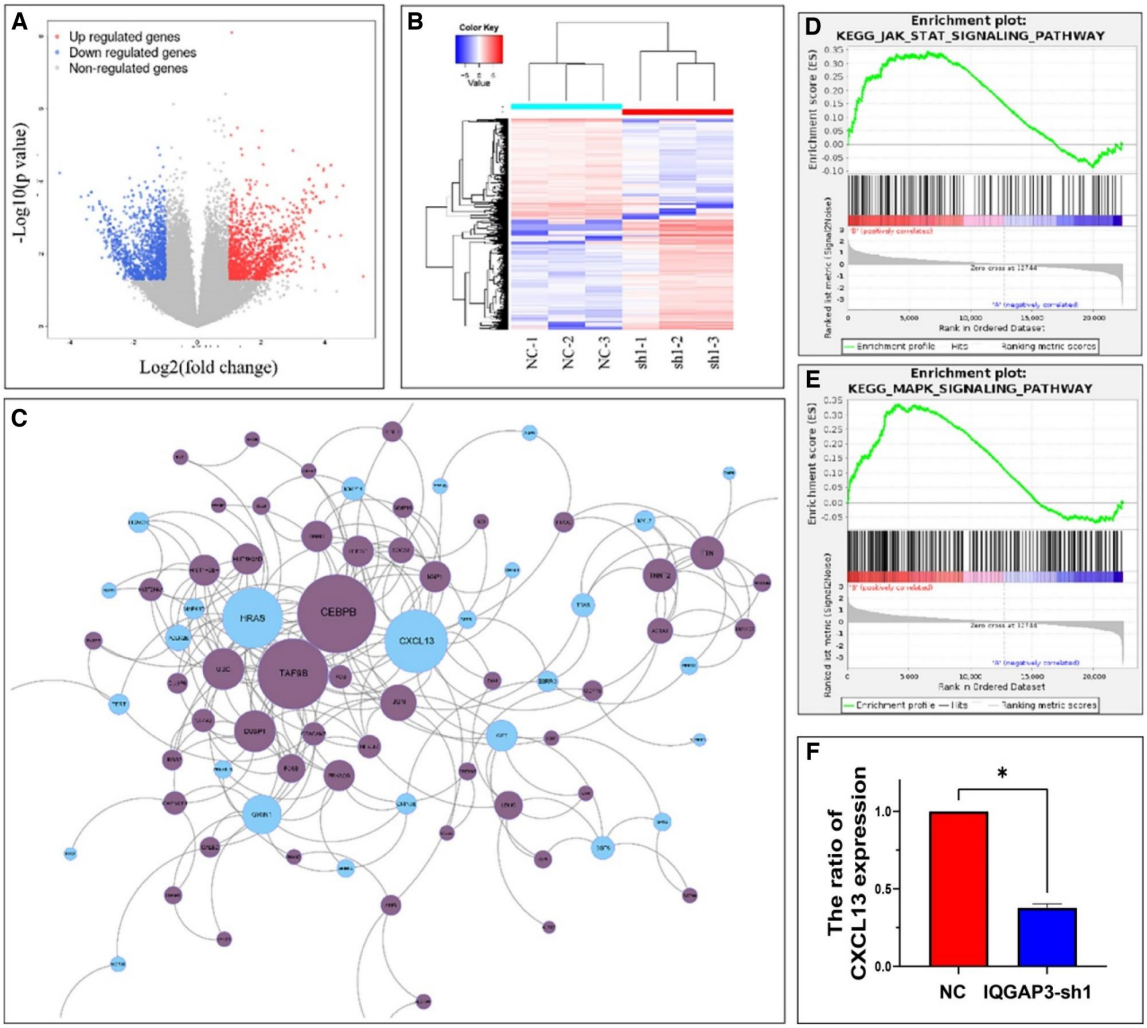
Results of cell mRNA sequencing and gene set enrichment analysis (GSEA) enrichment analysis. (A) Volcano plot of mRNA expression. (B) Heat map of DEG clusters. A total of 1,187 genes were downregulated, 1,286 genes were upregulated, and the expression of 32,870 genes did not change significantly. (C) After reducing IQGAP3 expression, the hub genes among the downregulated genes were CXCL13, HRAS, MAPK15, and MMP14, while the hub genes among the upregulated genes were mainly CEBPB, TAF9B, UBC, and DUSP1. (D, E) GSEA enrichment analysis revealed significant effects on the JAK–STAT pathway (*P* = 0.040) and the MAPK pathway (*P* = 0.015) following the knockdown of IQGAP3. (F) The concentration of CXCL13 in the conditioned medium of two groups of GC cells was detected by using an ELISA kit. The ELISA assay showed that CXCL13 secretion was significantly reduced in the IQGAP3-sh1 group (*P *< 0.001).

The CCLE database showed the expression level of IQGAP3 in various tumor cell lines (Figure 5A). The expression of IQGAP3 was measured in AGS, SGC-7901, BGC-823, HGC-27, NCI-N87, and GES-1 (Figure 5B). Three plasmids were then used to knock down the expression of IQGAP3 in the AGS cell lines. IQGAP3-sh1 exhibited the greatest knockout efficiency (Figure 5C and D). Transcriptome sequencing was performed on different groups of AGS cells. Volcano plots and heat maps were generated based on the differentially expressed genes (DEGs) in the two groups (Figure 6A and B). A protein–protein interaction network was plotted based on the DEGs (Figure 6C), with CXCL13 identified as the hub gene among the downregulated genes. The results showed that knocking down IQGAP3 probably decreased the activation of the JAK–STAT pathway (*P* < 0.05, Figure 6D) and the MAPK pathway (*P* < 0.05, Figure 6E). The ELISA assay showed that the concentration of CXCL13 decreased significantly in the conditioned medium of IQGAP3-sh1 GC cells (Figure 6F).

### Knocking down IQGAP3 expression inhibited the proliferation, migration, and invasion of GC cells

**Figure 7.**
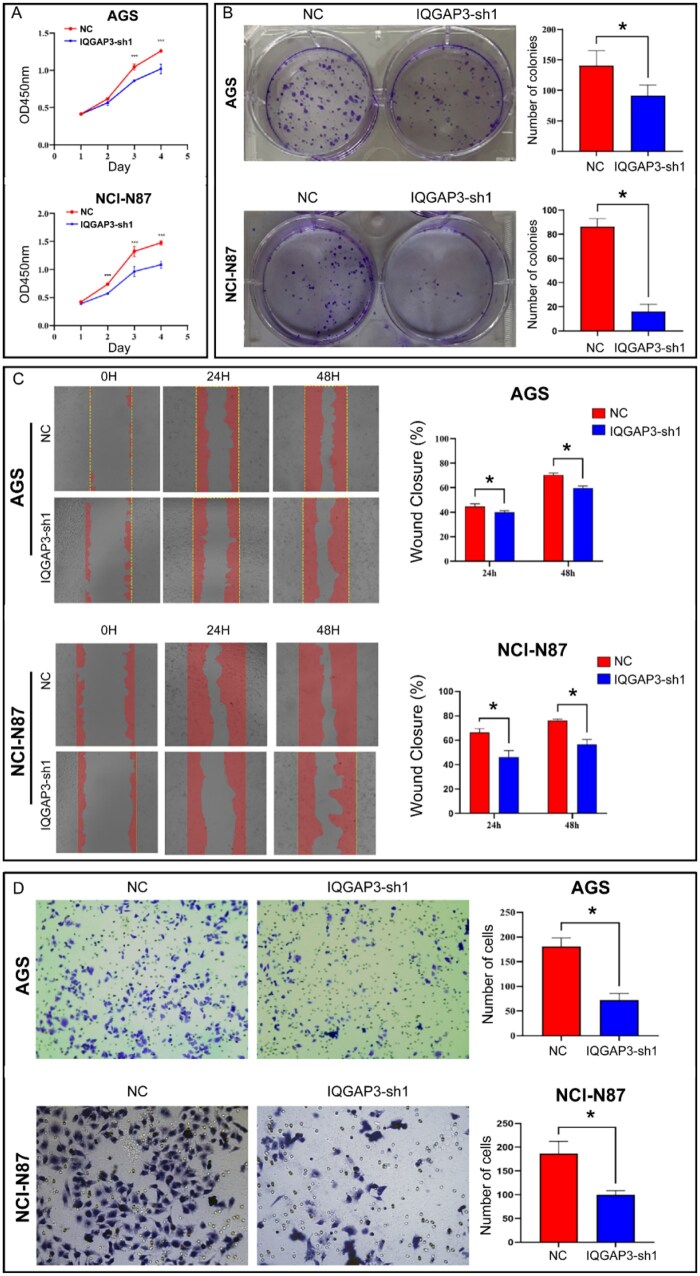
Effect of IQGAP3 on the proliferation, migration, and invasion of GC cells. (A) The ability of cells to proliferate was determined by using a CCK-8 assay. The results showed that knocking down IQGAP3 inhibited the proliferation of AGS cells. (*P* < 0.01). (B) The colony formation of AGC cells was inhibited by knocking down IQGAP3 (*P* < 0.01). (C) Cell-migration ability was determined by using a wound-healing migration assay. Knocking down IQGAP3 decreased the migration of AGC cells (*P* < 0.05). (D) Cell-invasion ability was determined by using the Matrigel invasion assay. Knocking down IQGAP3 decreased the invasion of AGC cells (*P* < 0.001). Cells in the migration and cell-invasion assays were counted by using Image J software. The assays were repeated three times for each group and plotted as bar graphs. *P < 0.05.

Compared with the NC group, the proliferation of IQGAP3-sh1 GC cells was decreased significantly (*P *< 0.05; Figure 7A). Similarly, the number of clones was significantly lower in the IQGAP3-sh1 group (*P *< 0.05, Figure 7B). The wound-healing migration assay revealed that IQGAP3-sh1 GC cells migrated less than did NC cells (*P *< 0.05, Figure 7C). Furthermore, the invasion of IQGAP3-sh1 GC cells was reduced significantly (*P *< 0.001, Figure 7D). Taken together, these results suggest that IQGAP3 plays an important role in the proliferation, migration, and invasion of GC cells.

### IQGAP3 modulated the expression of CXCL13 by regulating the activation of STAT3 and ERK pathways

**Figure 8.**
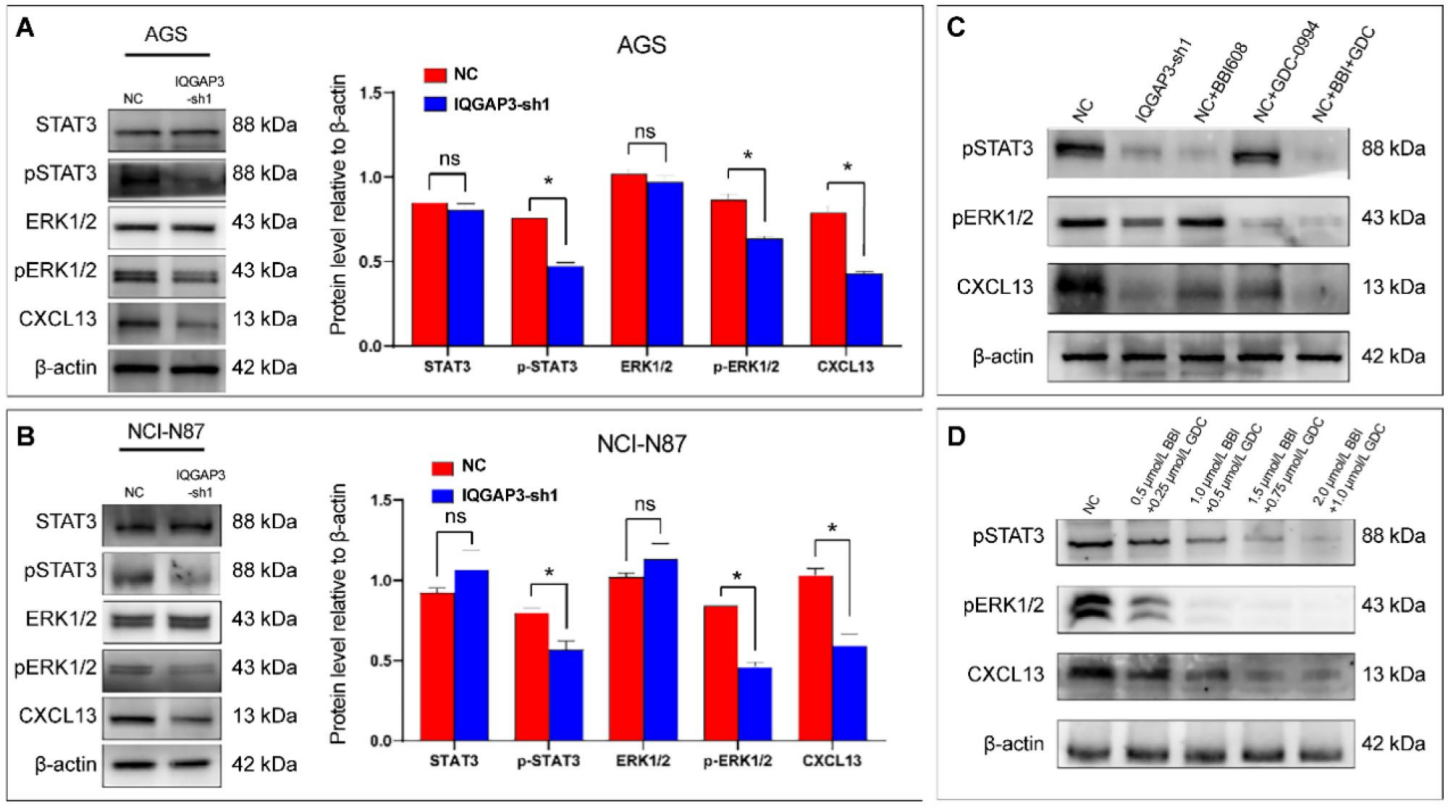
The expression of CXCL13 was decreased by IQGAP3 through the deactivation of STAT3 and ERK1/2. (A, B) Knockdown of IQGAP3 expression in the AGS and NCI-N87 cell lines decreased pSTAT3 activation (*P* < 0.05), pERK1/2 activation (*P* < 0.01), and CXCL13 expression (*P* < 0.01), while STAT3 and ERK1/2 expression remained unchanged. The experiment was repeated three times and a bar graph was plotted. (C) The pSTAT3 inhibitor (BBI608) and pERK1/2 inhibitor (GDC-0994) reduced the expression of CXCL13 significantly. (D) The expression of CXCL13 decreased in a concentration-dependent manner as the concentrations of BBI608 and GDC-0994 increased gradually. *P < 0.05.

To confirm the results of the transcriptome sequencing, we arranged Western-blotting assays. The result showed that the expression of CXCL13 was suppressed in the IQGAP3-sh1 group of AGS and NCI-N87 cells (*P *< 0.01, Figure 8A and B). Phosphorylation of pERK1/2 (*P *< 0.01) and pSTAT3 (*P *< 0.05) was also inhibited. However, no significant change in total ERK1/2 or STAT3 expression was observed. CXCL13 expression was reduced in the NC group following the single application of a pSTAT3 inhibitor (BBI608) or a pERK1/2 inhibitor (GDC-0994). Furthermore, the expression of CXCL13 decreased more significantly in the presence of both inhibitors (Figure 8C). Further experiments revealed that this phenomenon correlated with the concentration of the inhibitor used (Figure 8D). Taken together, these results suggested that IQGAP3 regulated the expression of CXCL13 by modulating the phosphorylation of pSTAT3 and pERK1/2.

### IQGAP3 modulated THP-1 cell differentiation to M0 macrophages by regulating CXCL13 secretion

**Figure 9.**
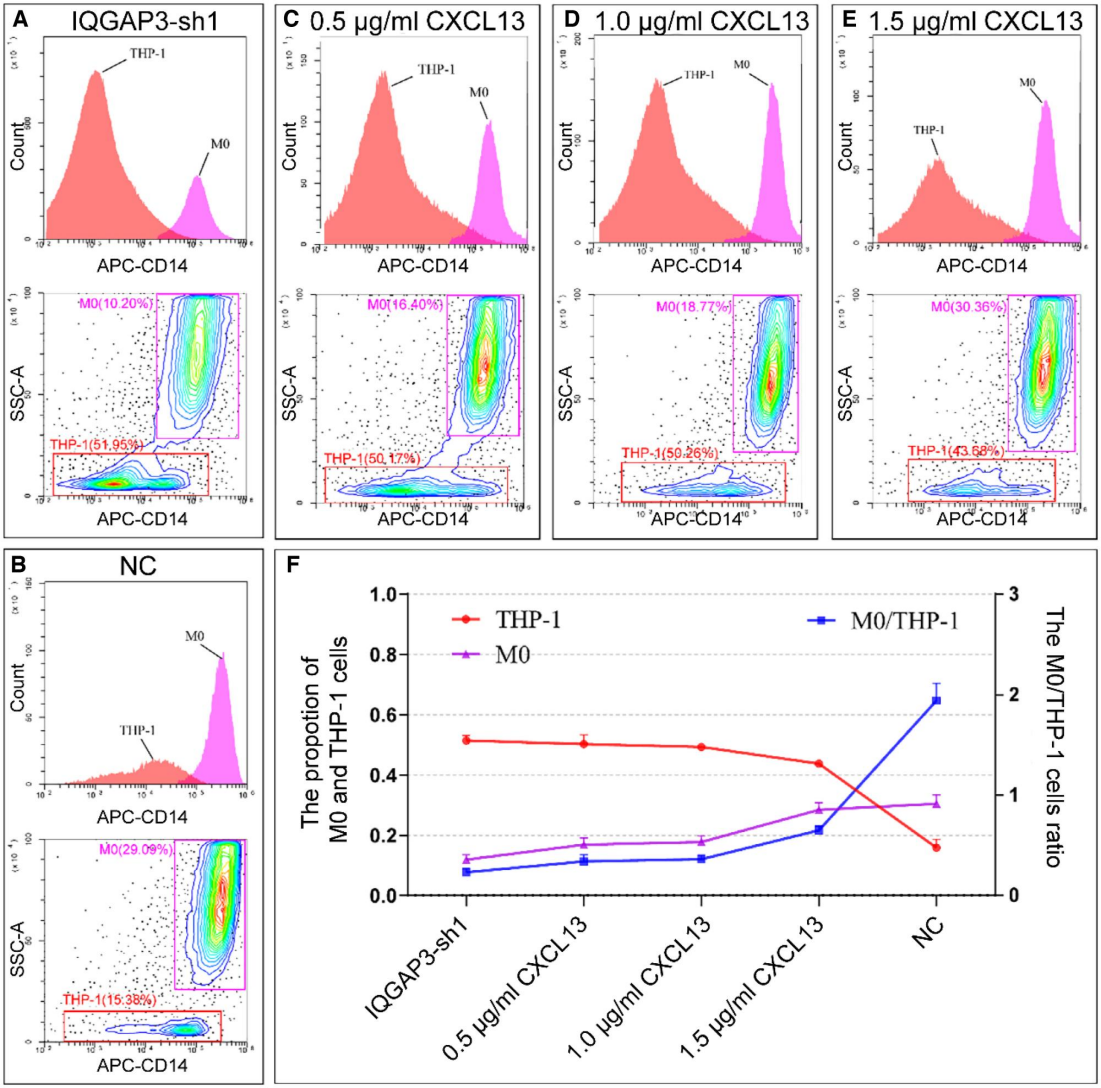
IQGAP3 modulated THP-1 cell differentiation to M0 macrophages by regulating CXCL13 secretion. (A) In the co-culture assay, THP-1 cells were cultured in conditioned medium from IQGAP3-sh1 AGS cells. The proportion of M0 macrophages was 10.20% and that of THP-1 cells was 51.95%. (B) In the NC group, the proportion of M0 macrophages was 29.09% and that of THP-1 cells was 15.38%. The result showed that knocking down IQGAP3 in AGS cells inhibited the proportion of M0 macrophages. (C) THP-1 cells were cultured in a conditioned medium from IQGAP3-sh1 AGS cells at a concentration of 0.5 μg/ml of rh-CXCL13. The proportion of M0 macrophages was found to be 16.40%, while the proportion of THP-1 cells was 50.17%. (D) When THP-1 cells were cultured in the same conditioned medium at a concentration of 1.0 μg/ml of rh-CXCL13, the proportion of M0 macrophages was 18.87%, while the proportion of THP-1 cells was 50.26%. (E) When the rh-CXCL13 concentration of conditioned medium was 1.5 μg/ml, the proportion of M0 macrophages was 30.36%, while the proportion of THP-1 cells was 43.68%. (F) With increasing concentrations of rh-CXCL13, the proportion of M0 macrophages increased, the proportion of THP-1 cells decreased, and the M0/THP-1 ratio increased. Flow-cytometry assays revealed that cells with higher SSC-A and APA-CD14 were M0 macrophages and those with lower SSC-A and APA-CD14 were undifferentiated THP-1 cells (labeled in red). All experiments were conducted in triplicate.

To further explore the regulatory role of IQGAP3 from GC cells on macrophages, THP-1 cells were cultured in conditioned medium from different groups of AGS cells with normal or reduced IQGAP3 expression. After 2 days of co-culture, the proportion of M0 macrophages/THP-1 cells was determined by using flow cytometry (Figure 9A and B). In the NC group, M0 macrophages accounted for 29.09% of the total, whereas, in the IQGAP3-sh1 group, they accounted for only 10.20%. These results implied that knocking down IQGAP3 expression significantly decreased the proportion of M0 macrophages. An increased concentration of rh-CXCL13 in the IQGAP3-sh1 cell medium (Figure 9C–E) resulted in an increased proportion of M0 macrophages and a decreased proportion of THP-1 cells (Figure 9F). No statistically significant difference was observed in the differentiation of M1 or M2 macrophages in the different IQGAP3 groups (*P *= 0.11 and *P *= 0.07, respectively) (Supplementary Figure 1).

### Knocking down IQGAP3 in AGS cells inhibited tumor growth in nude mice

**Figure 10.**
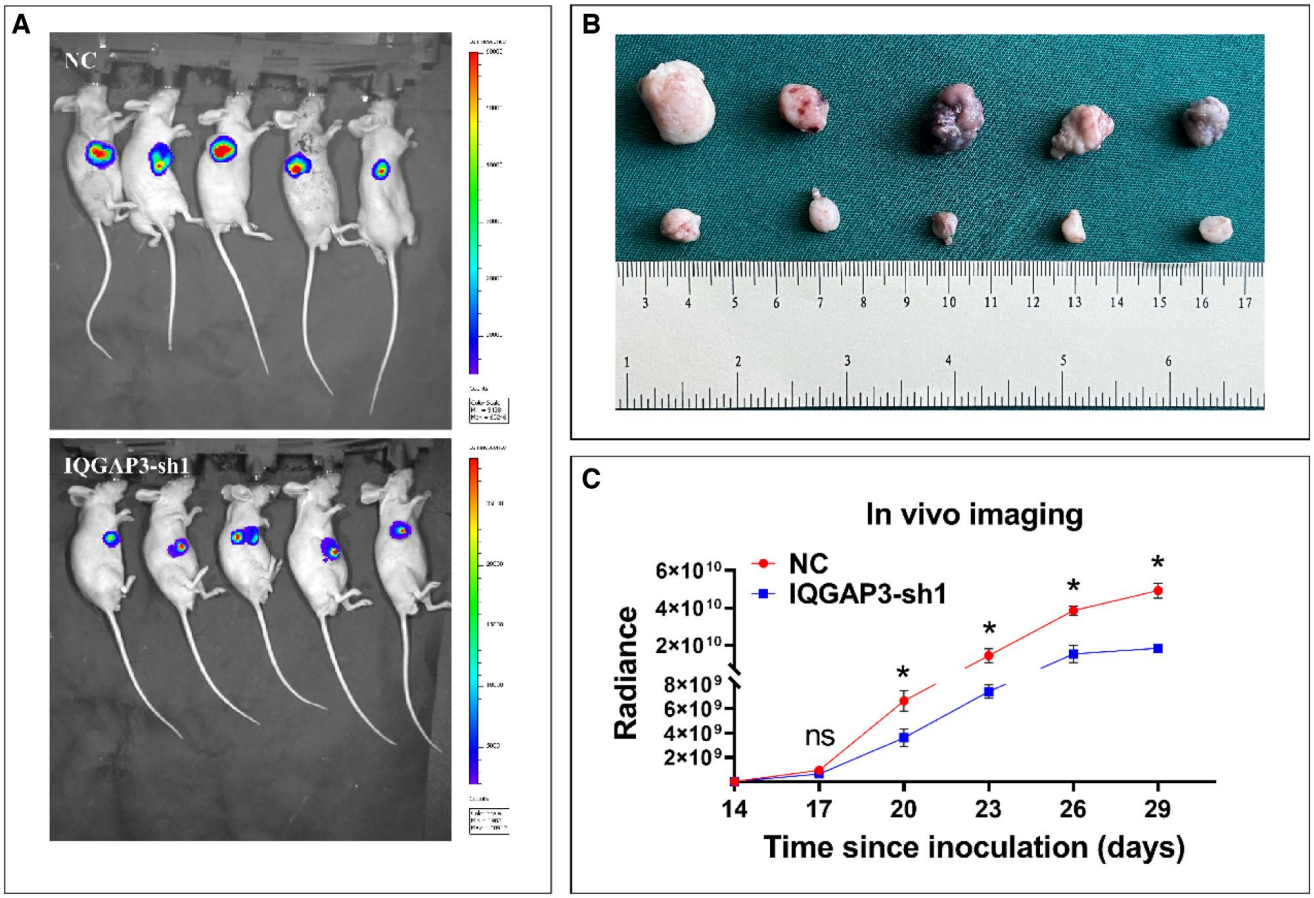
Subcutaneous tumor models in nude mice. (A) *In vivo* imaging showed that tumor growth was significantly reduced in the IQGAP3-sh1 group compared with the NC group. (B) Tumor volumes of the IQGAP3-sh1 and NC groups. (C) From Day 14 onwards, tumor size was measured by using *in vivo* imaging every 3 days and tumor growth curves were plotted. *P < 0.05.

**Figure 11.**
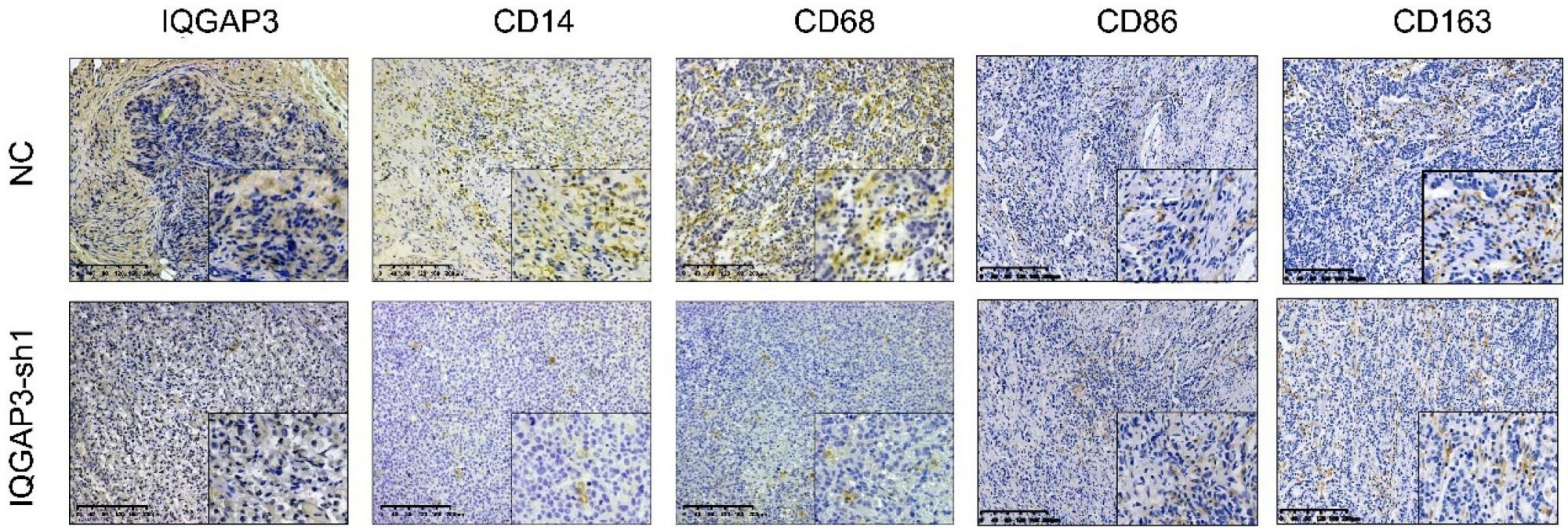
IHC staining of subcutaneous tumor sections. M0 macrophages were stained by using antibodies for the following surface biomarkers: CD14, CD68, CD86, and CD163. IHC staining showed that the proportion of M0 macrophages was significantly lower in the IQGAP3-sh1 group.

To further confirm the important role played by IQGAP3 in GC, we created a tumor model using nude mice *in vivo*. To better simulate the TME and observe the regulatory role of GC cells on macrophages, we mixed GC cells and THP-1 cells to construct tumors by subcutaneous injection in nude mice. *In vivo* experiments demonstrated that low IQGAP3 expression significantly reduced tumor growth (*P *< 0.05, Figure 10A and B). IHC analysis revealed that, compared with the NC group, low IQGAP3 expression was associated with decreased expression of CD14 and CD68. There was no significant change in the CD86 and CD163 expression (Figure 11).

## Discussion

IQGAP3 is considered a key regulator of metastasis and epithelial–mesenchymal transition, as it activates the transforming growth factor (TGF)-β signaling pathway in hepatocellular carcinoma (HCC) [16]. Besides, IQGAP3 is significantly correlated with microvascular invasion status and reduced survival in HCC [17]. In our study, the results showed that IQGAP3 promoted the proliferation of GC cells. It also promoted their clonogenicity and invasion.

**Figure 12.**
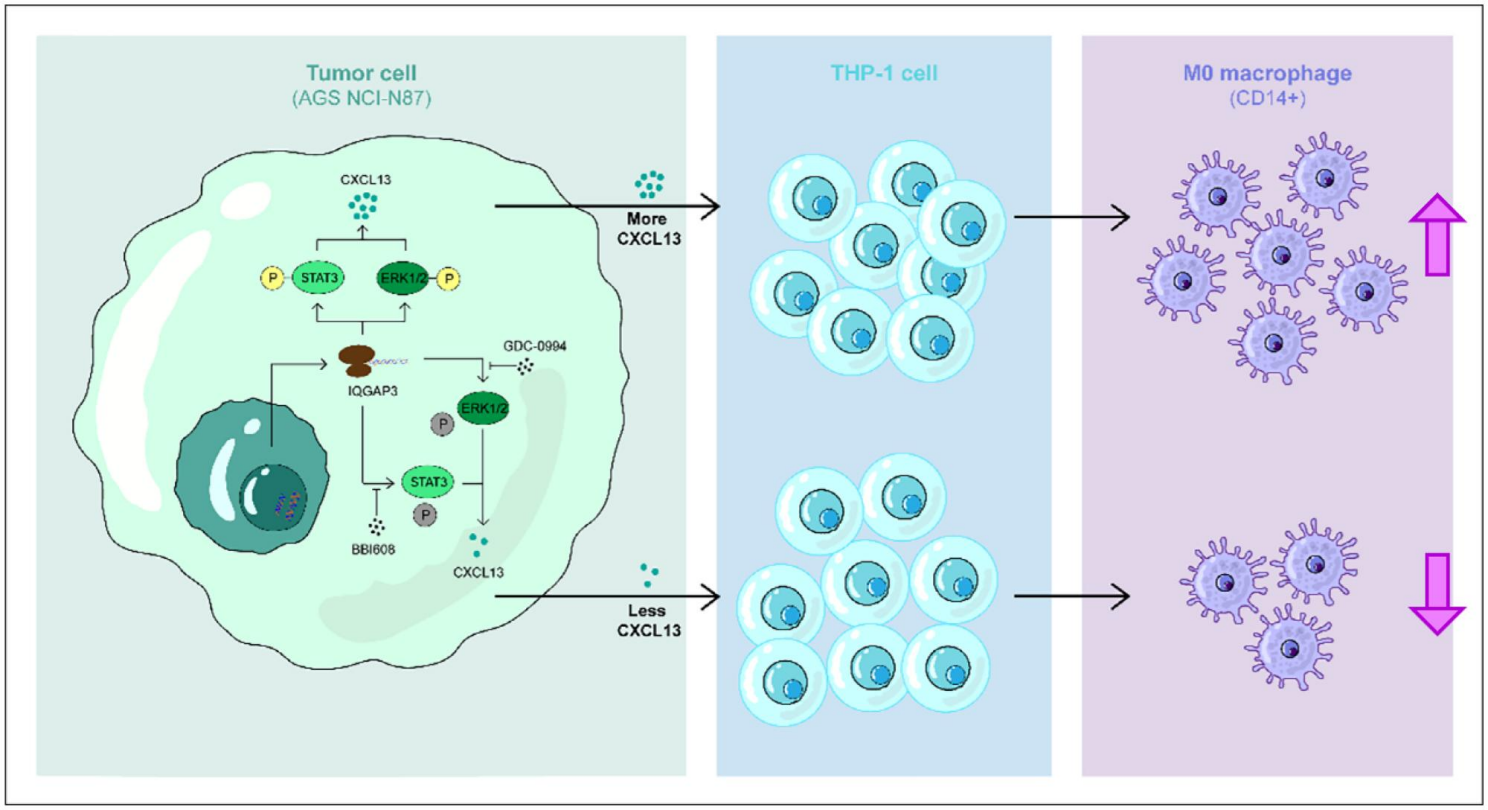
This is a schematic diagram showing how IQGAP3 regulates the secretion of CXCL13, which affects the proportion of M0 macrophages.

The IQGAP family plays a critical role in regulating multiple signaling pathways [18]. Nojima *et al.* first proposed that IQGAP3 was a key scaffolding protein in the Ras/ERK pathway, driving cell-cycle progression by stabilizing the Ras–GTP–Cyclin D1 axis [6]. Subsequent studies demonstrated that IQGAP3 mediated tumor progression in a variety of tumors [19]. Previous studies also highlighted the structural and functional similarities between IQGAP3 and IQGAP1 [17, 19]. IQGAP1 was first proven to form a signaling complex with MAPK, orchestrating a sequential phosphorylation process from Raf to ERK and modulating MAPK signaling [20, 21]. In GC, our study confirmed that IQGAP3 activated the ERK1/2 signaling pathway. Furthermore, we firstly discovered that IQGAP3 activated STAT3 simultaneously. Both the ERK1/2 and STAT3 signaling pathways played an important role in driving the expression and secretion of CXCL13 (Figure 12).

Currently, most studies have indicated that IQGAP3 functions as a promoter of tumorigenesis and is associated with a poor prognosis in various tumor types, including hepatocellular cancer, bladder cancer, lung cancer, and breast cancer [19, 22]. Our investigation analysed data from the TCGA and GEO databases. Data from the TCGA database did not indicate an association between high IQGAP3 expression and patient prognosis. However, the GEO database data showed a strong correlation between high IQGAP3 expression and a poorer prognosis in GC. Additionally, we performed IHC scoring on tumor tissue from 107 patients at our hospital. Survival analysis showed that patients with a high IQGAP3 level had a significantly worse prognosis. Therefore, we concluded that IQGAP3 was detrimental to the prognosis of patients with GC.

The development of GC is intricately linked to the TME—particularly macrophages [12]. The presence and behavior of TAMs, comprising M0, M1, and M2 macrophages, has a significant impact on various aspects of tumorigenesis, including tumor growth, angiogenesis, immune modulation, metastasis, and resistance to chemotherapy [23]. Previous studies on M1 and M2 macrophages showed that M1 macrophages promoted an antitumor immune response and inhibited tumor progression. Conversely, M2 macrophages promoted an immunosuppressive environment characterized by an increased expression of immunosuppressive molecules, such as PD-L1, IL-10, and TGF-β. They facilitate tumor immune evasion [24, 25].

Recent studies have emphasized the importance of M0 macrophages in tumor progression. Unlike M1 and M2 macrophages, which have more specialized roles in the TME, M0 macrophages have both pro-inflammatory and antitumor functions. Previous studies showed that a high infiltration of M0 macrophages was associated with a poor prognosis in various tumor types. For example, in gliomas, a high number of M0 macrophages is associated with poorer outcomes for patients [4]. The presence of M0 macrophages in hepatocellular cancer was associated with an unfavorable prognosis [26]. Additionally, an examination of the expression patterns of genes related to hypoxia in patients with hepatocellular cancer revealed a significant increase in M0 macrophages in two risk groups [27]. Interestingly, patients with endometrial cancer who were classified as high-risk also showed a significant increase in M0 macrophage abundance [28].

Transcriptomic analyses of ovarian cancer and glioblastoma have revealed that M0 macrophages have a transcriptional profile that is more similar to that of M2 macrophages [29, 30]. These findings highlight the important role of M0 macrophages in shaping the TME, thereby influencing cancer progression and patient outcomes [31]. In this study, we first proposed that IQGAP3 increased the infiltration of M0 macrophages into the TME of GC via a CXCL13-dependent mechanism. It implied that the infiltration of M0 macrophages was a potentially influential factor in the poor prognosis of GC patients. Studies of multiple myeloma have shown that CXCL13 levels are elevated in the blood and bone marrow of patients. Silencing CXCL13 was found to significantly reduce tumor growth and M2 macrophages [32]. However, no study of CXCL13 on M0 macrophages has yet been conducted.

Our experiments were also inadequate for distinguishing between different phenotypes of macrophages. Previous studies on macrophages mostly used the surface markers to distinguish between the different types of macrophages [33]. For example, in humans, M0 macrophages were labeled as CSF1R, CD14, CD68, CD11B, etc.; M1 macrophages were labeled as CD86, MARCO, and CXCL9; and M2 macrophages were labeled as TGM2, CD163, and CD206 [23]. However, macrophage polarization is a complex process, with current research focusing on the plasticity and functional continuity of macrophages [33]. Macrophages exist on a dynamic spectrum of phenotypes. These are precisely controlled by signals from the surrounding environment. These signals can switch rapidly [31]. The use of single-cell sequencing is a novel cell-labeling method that can be of great help in labeling TAMs [34]. In the future, our investigation will address current limitations in macrophage subtyping by using single-cell technologies and try to explore potential crosstalk between IQGAP3 and AKT–mTOR–STAT3 pathways to fully exploit its clinical potential.

## Conclusion

IQGAP3 is a critical regulator of GC that promotes tumor proliferation, clonogenicity, and invasive ability. Meanwhile, IQGAP3 remodels the immunosuppressive TME by increasing M0 macrophages via CXCL13-dependent mechanisms.

## Supplementary Material

goaf095_Supplementary_Data
