# Supplementary material for: High expression of IQGAP3 promotes the infiltration of M0 macrophages into the TME, resulting in a poor prognosis for gastric cancer patients
Source: Gastroenterol Rep (Oxf). 2025 Dec 23;13:goaf095. doi: 10.1093/gastro/goaf095 (PMC12724071; doi:10.1093/gastro/goaf095)
Supplement: goaf095_Supplementary_Data [file goaf095_supplementary_data.zip › TableS2.docx]

**Table S2. Genes positively associated with IQGAP3 and their functions.**

| **Gene** | **Gene description** | **Gene summary** |
| --- | --- | --- |
| **MFAP4** | microfibrillar-associated protein 4 | Bovine microfibril-associated protein related. Has affinities for both collagen and carbohydrates. |
| **ACKR1** | atypical chemokine receptor 1 | A glycosylated membrane protein, that serves as a non-specific receptor for multiple chemokines. Associated with the onset of malaria. |
| **MGP** | matrix Gla protein | Related to osteogenesis, chondrogenesis. (Keutel syndrome) |
| **SPARCL1** | SPARC-like 1 (hevin) | Function is unclear and may affect cellular synapse-related functions. |
| **SERPINF1** | serpin peptidase inhibitor, clade F (alpha-2 antiplasmin, pigment epithelium derived factor), member 1 | Inhibition of vascular survival associated with retinoblastoma differentiation. |
| **CCDC80** | coiled-coil domain containing 80 | Unclear function, glycoproteins of the extracellular matrix. |
| **AOC3** | amine oxidase, copper containing 3 | Monoamine oxidase, possibly related to diabetes. |
| **PRELP** | proline/arginine-rich end leucine-rich repeat protein | Associated with collagen adhesion. |
| **CYBRD1** | cytochrome b reductase 1 | Associated with iron absorption from food. |
| **TAGLN** | transgelin | An actin protein in smooth muscle. |
| **OGN** | osteoglycin | Trigger the formation of misplaced bone tissue with factor beta. |
| **PLN** | phospholamban | Important proteins in the myocardium. |
| **ASPN** | Asporin | Associated with inhibition of chondrogenesis. |
| **LMOD1** | leiomodin 1 (smooth muscle) | Associated with Graves'disease and thyroid-associated ophthalmopathy. |
| **FBLN1** | fibulin 1 | Associated with platelet adhesion. |
| **FHL1** | four and a half LIM domains 1 | Linked to Emery-Dreifuss muscular dystrophy in patients. |
| **SPON1** | spondin 1, extracellular matrix protein | Associated with intracellular protein formation. |
| **RBPMS2** | RNA binding protein with multiple splicing 2 | Involved in smooth muscle development in the digestive tract. |
| **NEXN** | nexilin (F actin binding protein) | Linked to dilated cardiomyopathy. |
| **C7** | complement component 7 | A critical element of the complement system. |
| **MYH11** | myosin, heavy chain 11, smooth muscle | A smooth muscle myosin from the myosin heavy chain family. |
| **CNN1** | calponin 1, basic, smooth muscle | Participate in inhibiting the proliferation of smooth muscle cells associated with vasculature. |
| **SYNPO2** | synaptopodin 2 | Facilitates alpha-actinin and filamin binding. |
| **SYNM** | synemin, intermediate filament protein | Playing a crucial role in providing structural support in muscle. |
| **CCL19** | chemokine (C-C motif) ligand 19 | Involved in regulating the immune system and processes related to inflammation. |
| **THBS4** | thrombospondin 4 | The gene encodes a protein that is a member of the thrombospondin protein family. Members of the Thrombospondin family are glycoproteins with adhesive properties that facilitate interactions between cells and the extracellular matrix. This protein oligomerizes into a pentamer and has the ability to interact with heparin and calcium. It is involved in local signaling in the developing and adult nervous system, and it contributes to spinal sensitization and neuropathic pain states. During the stromal response to invasive breast cancer，this gene becomes activated. It could also have an impact on inflammatory reactions in Alzheimer's disease. |
| **APOD** | Apolipoprotein D | linked with the enzyme lecithincholesterol acyltransferase. |
| **CFD** | complement factor D (adipsin) | Encodes a trypsin that is associated with digestion.. |
| **CXCL13** | chemokine (C-X-C motif) ligand 13 | B lymphocyte chemoattractant，known as Angie，is an antimicrobial peptide and CXC chemokine that is highly expressed in the follicles of the spleen，lymph nodes，and Peyer's patches. It specifically enhances the migration of B lymphocytes over T cells and macrophages by inducing calcium influx and chemotaxis in cells expressing Burkitt's lymphoma receptor 1(BLR-1).Consequently，it may play a crucial role in directing B lymphocytes towards follicles. |

Genes that are highly expressed in gastric cancer (marked in red) were CXCL13, ASPN and THBS4. Genes with low expression in gastric cancer were marked in green. Genes with no change in expression in gastric cancer were marked in gray.
