# Supplementary material for: High expression of IQGAP3 promotes the infiltration of M0 macrophages into the TME, resulting in a poor prognosis for gastric cancer patients
Source: Gastroenterol Rep (Oxf). 2025 Dec 23;13:goaf095. doi: 10.1093/gastro/goaf095 (PMC12724071; doi:10.1093/gastro/goaf095)
Supplement: goaf095_Supplementary_Data [file goaf095_supplementary_data.zip › TableS1.docx]

**Table S1. Results of co-expression analysis.**

| **Gene expression levels in GC**  **(29)** | **Up-regulated**  **(3)** | **Down-regulated**  **(12)** | **No sig.**  **(14)** |
| --- | --- | --- | --- |
| **Genes** | **CXCL13**, ASPN, THBS4 | ACKR1, PRELP, LMOD1, FHL1, RBPMS2, C7, MYH11,CNN1, SYNPO2, SYNM, APOD, CFD | MFAP4, MGP, SPARCL1, SERPINF1, CCDC80, AOC3, CYBRD1, TAGLN, OGN, PLN, FBLN1, SPON1, MEXN, CCL19 |
